# Advances in the application of multi-omics in tumor immunotherapy

**DOI:** 10.3389/fgene.2026.1787895

**Published:** 2026-04-20

**Authors:** Tongtong Zhang, Quan-Yong Luo, Yunyun Zhu

**Affiliations:** Department of Nuclear Medicine, Shanghai Jiao Tong University Affiliated Sixth People’s Hospital, Shanghai, China

**Keywords:** biomarkers, multi-omics, precision medicine, radiomics, tumor immunotherapy

## Abstract

As a pivotal therapeutic approach following surgery, chemoradiotherapy, and molecular targeted therapy, tumor immunotherapy has revolutionized survival outcomes for cancer patients, with immune checkpoint inhibitors (ICIs) demonstrating remarkable efficacy in clinical practice. However, challenges such as Immunotherapy resistance and significant individual variability in response persist, underscoring the critical need for precise tumor assessment and identification of benefit populations to achieve precision and personalization in immunotherapy. Multi-omics technologies, by integrating multidimensional data from genomics, transcriptomics, proteomics, metabolomics, and radiomics, enable comprehensive analysis of tumor development mechanisms, tumor microenvironment characteristics, and immunotherapy response patterns at molecular, cellular, tissue, and systemic levels. This review systematically examines the current applications, clinical value, and future prospects of multi-omics in tumor immunotherapy, with a focus on the development and utilization of radiomics in immunotherapy efficacy evaluation and prognostic prediction, thereby providing theoretical foundations and technical support for the precise implementation of tumor immunotherapy.

## 1 Overview of tumor immunotherapy

Tumor immunotherapy achieves tumor control or even specific eradication by overcoming tumor immune evasion mechanisms, reversing the immunosuppressive state or overcoming immunosuppressive mechanisms, and restoring and enhancing the body’s anti-tumor immune response (Kim et al., 2025). Compared with traditional surgical, chemoradiotherapy, and chemotherapy, tumor immunotherapy exhibits significant advantages such as high specificity and sustained efficacy, and has been widely applied in the treatment of various malignant tumors.

Tumor immunotherapy primarily includes immune checkpoint inhibitors (ICIs), adoptive cell immunotherapy, and cancer vaccines (Zhao et al., 2025), among which ICIs are currently a major research focus. Based on different target molecules, clinically applied ICI are mainly classified into three categories: cytotoxic T-lymphocyte antigen 4 (CTLA-4) monoclonal antibodies, programmed cell death 1 (PD-1) monoclonal antibodies, and programmed cell death ligand 1 (PD-L1) monoclonal antibodies (Helmink et al., 2020). Currently, approved ICI therapies for malignant tumors include malignant melanoma, non-small cell lung cancer (NSCLC), renal cell carcinoma, bladder cancer, head and neck squamous cell carcinoma, Hodgkin’s lymphoma, gastric cancer, and hepatocellular carcinoma (Cabrita et al., 2020). In China, 17 ICI have been approved for marketing, including 10 PD-1 inhibitors (representative drugs: pembrolizumab, nivolumab), 5 PD-L1 inhibitors (representative drugs: durvalumab, atezolizumab), 1 CTLA-4 inhibitor (ipilimumab), and 1 PD-L1/CTLA-4 bispecific antibody (cadunimab) (Petitprez et al., 2020).

Tumor immunotherapy has fundamentally reshaped cancer treatment by restoring and amplifying host antitumor immune responses. Despite the remarkable clinical success of immune checkpoint inhibitors (ICIs), only a subset of patients achieve durable benefit, and both primary and acquired resistance remain major clinical challenges. These limitations raise a central scientific question: how can multidimensional biological data be integrated to achieve precise patient stratification and individualized immunotherapy decision-making? Traditional predictive strategies based on single biomarkers, such as PD-L1 expression or tumor mutational burden (TMB), provide limited accuracy due to tumor heterogeneity and the dynamic complexity of the tumor microenvironment (TME). Multi-omics approaches integrating genomics, transcriptomics, proteomics, metabolomics, immunomics, and radiomics offer a comprehensive framework to address this challenge and enable precision immunotherapy.

In this context, understanding the mechanisms underlying resistance and inter-patient heterogeneity has become a critical priority. Some patients develop primary or acquired resistance to immunotherapy, and significant inter-patient variability in therapeutic response severely impacts efficacy evaluation and prognostic assessment (Pirouzbakht et al., 2025). Moreover, the interaction mechanisms between ICI and the tumor microenvironment (TME) are not fully elucidated, and the complexity of the TME poses difficulties for precise immunotherapy implementation (Gao F. et al., 2025). Therefore, comprehensive analysis of tumor immune characteristics using multi-omics technologies is crucial for optimizing immunotherapy strategies and enhancing treatment outcomes.

A structured literature search was conducted using PubMed and Web of Science databases. The search terms included combinations of “multi-omics,” “tumor immunotherapy,” “immune checkpoint inhibitors,” “radiomics,” “spatial transcriptomics,” and “immune microenvironment.” Studies published between 2015 and 2025 were prioritized, with emphasis on peer-reviewed clinical and translational research. Conference abstracts were reviewed selectively, and high-quality original research and systematic studies were preferentially included to ensure evidence robustness.

## 2 Immune checkpoints and their mechanisms of action

**FIGURE 1:**
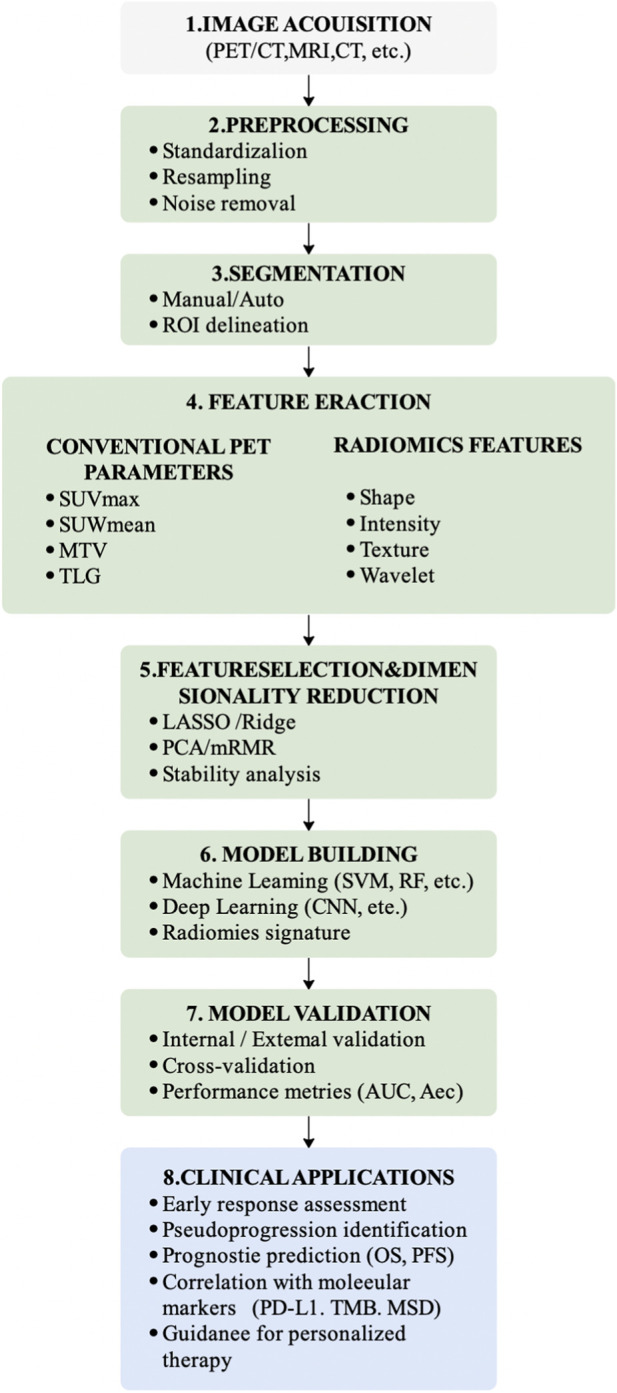
Multi-omics framework for precision immunotherapy.

Immune checkpoints are specific receptors expressed on the surface of immune cells (Figure 1), which regulate immune cell activity upon binding with their corresponding ligands (Guo et al., 2025). Under physiological conditions, immune checkpoints participate in maintaining immune system homeostasis, preventing excessive immune responses from causing damage to normal tissues. However, tumor cells can exploit the immune checkpoint mechanism to achieve immune evasion by expressing immune checkpoint ligands, binding to immune checkpoints on the surface of immune cells, and suppressing immune cell activity, thereby evading attacks by immune cells (Lu X. et al., 2025).

T cells are the core force of antitumor immunity, and their activation requires two key signals: first, the binding of T cell receptors to major histocompatibility antigens and homologous peptide complexes on antigen-presenting cells; second, the interaction between co-stimulatory receptor CD28 and B7 ligands on antigen-presenting cells (Hegde and Chen, 2020). Upregulation of inhibitory immune checkpoints suppresses T cell activation, among which CTLA-4 and PD-1 are the most thoroughly studied inhibitory immune checkpoint molecules (Zhou et al., 2025).

CTLA-4 is a transmembrane protein highly expressed on the surface of activated CD4^+^ and CD8^+^ T cells as well as regulatory T cells, and is a homolog of CD28 (Gao Q. et al., 2025). Compared to CD28, CTLA-4 exhibits higher affinity for the B7 ligand on the surface of antigen-presenting cells. The binding of CTLA-4 to B7 ligand can block the co-stimulatory effect of CD28 on T cells, thereby inhibiting T cell activation (Thorsson et al., 2018). PD-1 is primarily induced on the surface of immune cells such as activated T cells, B cells, natural killer cells, and dendritic cells. Its ligand, PD-L1, is mainly expressed on the surface of tumor cells in diseases such as non-small cell lung cancer (NSCLC), melanoma, gastric cancer, bladder cancer, and ovarian cancer (Le et al., 2025). After T cell activation, PD-1 expression increases. Binding to PD-L1/PD-L2 can inhibit the activation of downstream signaling pathways, thereby suppressing the activation, proliferation, and antitumor function of tumor antigen-specific CD8^+^ T cells, leading to tumor immune evasion (Wang Y. et al., 2025). The core mechanism of immune checkpoint inhibitor (ICI) therapy is to block these inhibitory signals, thereby restoring and enhancing the body’s antitumor immune response (Lu C. et al., 2025).

## 3 Core applications of multi-omics in tumor immunotherapy

**FIGURE 2:**
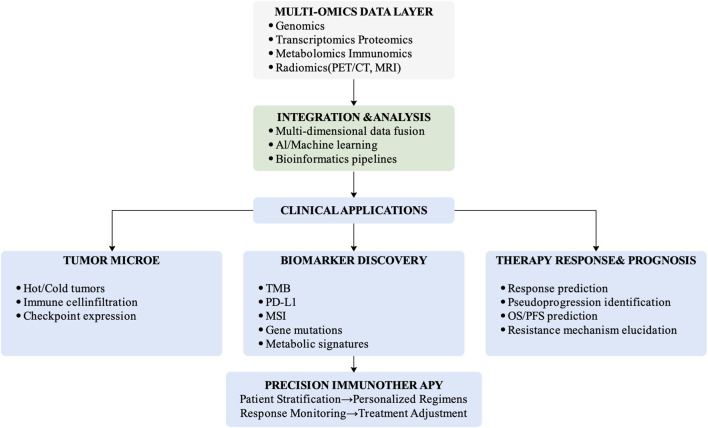
Standard radiomics workflow for immunotherapy assessment.

Multi-omics integration extends beyond parallel analysis of individual datasets and aims to construct cross-scale biological models (Figure 2). Horizontal integration combines datasets at the same biological layer, whereas vertical integration links molecular alterations with higher-order phenotypes such as tissue architecture and imaging signatures.

Advanced computational frameworks have been developed to facilitate multi-omics integration, including matrix factorization–based methods such as MOFA (Multi-Omics Factor Analysis), clustering algorithms such as iCluster, and deep-learning–based multimodal fusion models. These approaches enable joint dimension reduction, latent factor extraction, and predictive modeling across heterogeneous datasets, thereby improving biomarker robustness and generalizability.

### 3.1 Genomics and transcriptomics

The application of genomics and transcriptomics in tumor immunotherapy primarily focuses on biomarker identification, elucidation of resistance mechanisms, and discovery of therapeutic targets (Jeong et al., 2025). From a genomic perspective, technologies such as whole-genome sequencing and whole-exome sequencing enable the detection of tumor-related gene mutations, copy number variations (CNVs), and gene fusions, which are closely associated with tumor immunogenicity and response to immune checkpoint inhibitors (ICIs) (Zhang Z. et al., 2025)^.^ Tumor mutational burden (TMB) remains one of the most extensively studied genomic biomarkers, with higher TMB generally correlating with improved response to ICIs due to increased neoantigen load (Xie et al., 2025). Similarly, mismatch repair deficiency (dMMR) and high microsatellite instability (MSI-H) enhance tumor immunogenicity and have been established as predictive markers for immunotherapy efficacy across multiple tumor types (Zhang C. C. et al., 2025).

Beyond global mutational metrics, specific genomic alterations have been shown to influence immunotherapy response. For example, STK11 (LKB1) mutations in non-small cell lung cancer are associated with reduced CD8^+^ T-cell infiltration and an immune “cold” tumor phenotype, leading to poor response to PD-1 blockade (Li et al., 2025). KEAP1 mutations are likewise linked to inferior outcomes and resistance to ICIs through dysregulation of oxidative stress pathways and immune signaling. In addition, defects in antigen presentation pathways, such as B2M loss, and alterations in IFN-γ signaling components further contribute to primary or acquired resistance. These findings underscore that genomic biomarkers should be interpreted within the broader context of tumor–immune interactions rather than as isolated predictors (Dong et al., 2025).

Transcriptomic profiling provides complementary functional insight into tumor–immune dynamics. RNA sequencing enables comprehensive analysis of immune-related gene expression patterns and immune cell states within the tumor microenvironment (Zander et al., 2025). Immune gene expression signatures, particularly the T cell–inflamed profile and interferon-γ–associated signatures, have demonstrated predictive value for immunotherapy response across various tumor types. Such transcriptomic signatures facilitate classification of tumors into immune-inflamed, immune-excluded, and immune-desert phenotypes, thereby improving patient stratification and therapeutic decision-making.

### 3.2 Proteomics and metabolomics

Proteomics enables direct assessment of immune-related protein expression and signaling activity (Lim et al., 2025). In addition to tissue-based quantification of PD-1, PD-L1, and CTLA-4, increasing attention has been directed toward circulating protein biomarkers, including soluble PD-L1 (sPD-L1), inflammatory cytokine panels (e.g., IL-6, IFN-γ), and exosome-associated immune proteins (Wang D. et al., 2025). These blood-based biomarkers provide minimally invasive tools for dynamic monitoring of immunotherapy efficacy and immune-related adverse events, enhancing longitudinal assessment capability (Chen et al., 2025).

Metabolomics focuses on the alterations of tumor metabolites. The abnormal metabolism of tumor cells not only provides energy and material basis for their own proliferation but also affects the immune function of the tumor microenvironment (Murillo et al., 2025). Tumor metabolic reprogramming plays a critical role in immune suppression. Lactate accumulation resulting from aerobic glycolysis acidifies the tumor microenvironment, impairing CD8^+^ T-cell cytotoxic function and promoting regulatory T-cell expansion (Baek et al., 2025). Additionally, activation of the IDO–tryptophan–kynurenine pathway depletes tryptophan and induces T-cell anergy, contributing to immune escape and therapeutic resistance (Zhang X. et al., 2025). Metabolomic profiling therefore provides mechanistic insight into immunotherapy resistance and identifies potential combinational therapeutic targets (Rosario et al., 2024).

### 3.3 Immunomics and immune profiling

Immunomics primarily investigates the composition, function, and interactions of immune cells within the tumor microenvironment. Through techniques such as flow cytometry and single-cell sequencing, it enables comprehensive analysis of the distribution characteristics, phenotypic changes, and gene expression profiles of immune cell subsets (Xie et al., 2024). For instance, the infiltration level and functional status of CD8^+^ T cells are closely associated with the efficacy of ICI therapy, with patients exhibiting abundant functional CD8^+^ T cell infiltration often demonstrating better therapeutic outcomes (Tang et al., 2024)^.^ Additionally, alterations in the proportions of immunosuppressive cells such as regulatory T cells and macrophages can influence Immunotherapy responses. Immunomics can precisely quantify changes in these cell subsets, providing a basis for adjusting treatment regimens (Prakadan et al., 2021).

The advancement of single-cell sequencing technology has revolutionized immunomics research, enabling the revelation of immune cell heterogeneity at the single-cell level and the discovery of rare immune cell subpopulations and functional states (Wang et al., 2024). Through single-cell sequencing, dynamic changes in immune cell subpopulations during immunotherapy can be thoroughly analyzed, elucidating the molecular mechanisms underlying immune cell activation or exhaustion, thereby providing critical insights for the development of novel immunotherapy strategies (Xu et al., 2024).

Multi-omics approaches have provided important insights into mechanisms of primary and acquired resistance to immunotherapy. Genomic alterations such as B2M loss impair antigen presentation, while mutations in the IFN-γ signaling pathway disrupt immune activation. STK11 and KEAP1 mutations are associated with immune-cold phenotypes and poor ICI response in NSCLC. Transcriptomic analyses reveal exhausted T-cell signatures and immune-excluded states, whereas metabolomic profiling identifies lactate accumulation and tryptophan depletion as mediators of immune suppression. Integrating these omics layers allows mechanistic mapping of resistance pathways and may guide combination therapeutic strategies.

Recent advances in spatial transcriptomics and single-cell multi-omics technologies have further enabled high-resolution mapping of tumor–immune interactions. These approaches identify spatially distinct immune niches, tertiary lymphoid structures, and immune-excluded phenotypes that are not captured by bulk sequencing. Such spatially resolved data improve understanding of intratumoral heterogeneity and may enhance predictive modeling of immunotherapy response.

## 4 Applications and development of imaging omics in tumor immunotherapy

Radiomics involves a standardized analytical workflow, including image acquisition and preprocessing, tumor segmentation, quantitative feature extraction (first-order statistics, shape descriptors, and texture features such as GLCM and GLRLM), feature selection (e.g., LASSO, mRMR), and predictive modeling using machine-learning classifiers such as support vector machines, random forest, gradient boosting algorithms, or deep neural networks (Cristescu et al., 2018). Standardization of imaging protocols and feature harmonization across institutions remain critical challenges for reproducibility and clinical translation (Grehaigne et al., 2024).

Radiomics, by extracting metabolic parameters and texture features from PET/CT images, provides a more comprehensive assessment of tumor biology and treatment response.

For example, higher entropy or skewness values may correspond to spatial immune heterogeneity or uneven metabolic activity. Bridging imaging phenotypes with underlying immunobiology remains an essential research priority.

### 4.1 Application of imaging omics in evaluation of immunotherapy efficacy

Traditional tumor efficacy evaluation criteria, such as the Response Evaluation Criteria in Solid Tumors (RECIST) 1.1, primarily rely on changes in tumor lesion size. However, due to the unique mechanisms of immune checkpoint inhibitor (ICI) therapy, special response patterns such as delayed response, pseudoprogression, and dissociation reactions may occur during treatment, making it difficult for conventional standards to accurately assess efficacy. Imagingomics, by extracting metabolic parameters and texture features from PET/CT images, can more comprehensively reflect the biological characteristics of tumors and treatment responses, providing a more precise basis for efficacy evaluation (Quek et al., 2024).

18F-FDG PET/CT is currently the most widely used PET imaging technique, with its radiomics features including semi-quantitative parameters, volume-related parameters, and texture characteristics (Glass et al., 2024). These features are closely associated with tumor metabolic activity, heterogeneity, and immune microenvironment, enabling effective evaluation of early therapeutic efficacy in ICI treatment (Zhang et al., 2024). For instance, studies have found that SUVmax in patients with effective ICI treatment significantly decreases early in the treatment course (after 8 weeks), while MTV and TLG show significant changes only in the mid-treatment phase, suggesting that radiomics parameters can predict treatment response at an early stage. A study of 45 lymphoma patients demonstrated that risk stratification based on 18F-FDG PET/CT radiomics features was strongly correlated with 2-year survival rates, with the low-risk group achieving a 100% 2-year survival rate compared to only 53% in the high-risk group.

Furthermore, radiomics can effectively identify specific response patterns in ICI therapy (Tan et al., 2024)^.^ Pseudoprogression, a common phenomenon in ICI treatment, manifests as initial tumor lesion enlargement or increased metabolism followed by gradual tumor burden reduction, which traditional imaging methods may misinterpret as disease progression. Radiomics distinguishes pseudoprogression from true progression by analyzing tumor texture characteristics and metabolic heterogeneity parameters (Minegishi et al., 2024). For instance, the developed PET radiotracer 68Ga-grazytracer targeting granzyme B exhibits radiomics features that specifically detect the functional status of effector T cells, effectively differentiating true tumor progression from pseudoprogression. Dissociation response, characterized by partial tumor regression with other lesions progressing, can be promptly identified by radiomics due to its high sensitivity and systemic evaluation capability, providing a basis for adjusting treatment regimens. However, it should be acknowledged that current evidence supporting radiomics-based differentiation of pseudoprogression is largely derived from retrospective studies with limited sample sizes. Prospective multicenter validation is required before routine clinical adoption.

Although 18F-FDG PET/CT remains a dominant imaging modality in immunotherapy evaluation, CT-based and MRI-based radiomics have also demonstrated predictive value. CT-derived texture features have been associated with tumor heterogeneity and immune infiltration patterns, while MRI-based radiomics provides functional and diffusion-related parameters reflecting tumor cellularity and microenvironment complexity. Multimodal imaging integration may therefore enhance predictive robustness compared to single-modality approaches.

### 4.2 Application of imaging omics in prognostic prediction of immunotherapy

Imaging biomarkers can reflect the intrinsic biological characteristics and immune microenvironment status of tumors, serving as a critical tool for predicting the prognosis of immunotherapy in cancer patients (Cheng et al., 2024). Multiple studies have demonstrated that 18F-FDG PET/CT imaging biomarkers are closely associated with overall survival (OS) and progression-free survival (PFS) in patients (Li et al., 2024). For instance, a study on melanoma patients treated with ipilimumab revealed that those with high baseline metastatic tumor volume (MTV) had significantly shorter median OS compared to those with low MTV, indicating that MTV can serve as an independent prognostic predictor. Additionally, research has confirmed that melanoma patients with baseline MTV ≥8.2 cm^3^ exhibited lower survival rates after anti-PD-1 therapy, which was identified as an independent prognostic factor for mortality.

In addition to traditional metabolic and volumetric parameters, radiomics texture features also hold significant value in prognostic prediction (Liu et al., 2021). Texture features can reflect tumor heterogeneity, which is closely associated with tumor invasiveness and immune evasion capacity (Cao et al., 2023). Studies on advanced NSCLC patients receiving ICI therapy have found that higher skewness heterogeneity (reflecting tumor lesion asymmetry) and kurtosis heterogeneity (indicating lesion irregularity) are associated with greater disease progression and poorer prognosis. The visualization of tumor infiltration CD8^+^ T cell exhaustion status through PET radiomics methods can effectively predict ICI treatment efficacy, providing crucial reference for patient prognosis assessment.

### 4.3 Application of imaging omics in molecular typing of tumors and beneficiary population screening

Tumor molecular profiling serves as the foundation for precision immunotherapy. As a non-invasive classification tool, radiomics can indirectly reflect the molecular expression status of tumors through imaging characteristics (Zhang et al., 2022). PD-L1 expression level is a critical biomarker for screening patients who may benefit from ICI therapy. Multiple studies have demonstrated that 18F-FDG PET/CT radiomics features are closely associated with PD-L1 expression levels, enabling non-invasive prediction of PD-L1 expression status (Zhang et al., 2023). For instance, a study involving 255 NSCLC patients extracted radiomics features from 18F-FDG PET/CT images and constructed a combined model. The results revealed that 18 features were valuable for predicting PD-L1 expression>1%, while 7 features were valuable for predicting PD-L1 expression>50%. This model exhibited good predictive performance.

Furthermore, radiomics can be utilized to predict genomic features such as tumor mutation burden and microsatellite instability, providing a basis for patient stratification (BMJ Publishing Group Ltd., 2023). For instance, a phase III clinical trial demonstrated that composite radiomics features predicted better overall survival (OS) in melanoma patients treated with pembrolizumab compared to the RECIST standard, effectively screening out the benefit population. In esophageal cancer patients, baseline 18F-FAPI-04 PET/CT images revealed correlations between the mean SUV, peak SUV, and PD-L1 expression, offering a novel approach for non-invasive assessment of PD-L1 expression.

Beyond PD-L1 expression, radiomics-based models have also been explored for non-invasive prediction of tumor mutational burden (TMB), microsatellite instability (MSI), and immune infiltration scores, further expanding the framework for beneficiary population screening.

### 4.4 Prospects and challenges in imagingomics

With the continuous advancement of artificial intelligence technology, the application prospects of radiomics in tumor immunotherapy are promising (Song et al., 2023). Multimodal radiomics, by integrating various imaging data such as PET/CT, MRI, and CT, can more comprehensively reflect the biological characteristics of tumors, thereby further improving the accuracy of therapeutic efficacy evaluation, prognosis prediction, and molecular subtyping (Shao et al., 2023). For example, combining the metabolic information from PET/CT with the functional information from MRI enables more precise assessment of the immune status of the tumor microenvironment (Li et al., 2023). Additionally, the integrated analysis of radiomics with other omics data, such as genomics, transcriptomics, and proteomics, can construct multidimensional predictive models, providing more comprehensive support for the precision and individualization of tumor immunotherapy (Chen et al., 2022).

However, radiomics still faces numerous challenges in clinical application (Peng et al., 2020). Firstly, differences in imaging equipment, scanning parameters, and image reconstruction methods among various medical institutions lead to prominent issues of reproducibility and standardization of radiomics features, which hinder their clinical promotion and application. Secondly, most current radiomics studies are small-sample retrospective analyses, and the reliability and generalizability of the results require validation through large-sample prospective studies (Heumos et al., 2022). Additionally, the biological significance of radiomics features remains unclear, and how to effectively correlate radiomics features with the pathophysiological mechanisms and immune function status of tumors remains a question requiring further in-depth research (Virgile et al., 2022). In the future, the clinical application of radiomics in tumor immunotherapy is expected to be further advanced through the establishment of unified imaging standards, the conduct of large-sample multicenter clinical trials, and the strengthening of interdisciplinary integration between radiomics and other fields (Hu et al., 2022).

A major challenge of AI-driven radiomics models is their “black-box” nature, which may limit clinical trust and regulatory acceptance. Emerging explainable AI approaches, including SHAP (Shapley Additive Explanations) and feature importance visualization, allow interpretation of model outputs and enhance transparency. Improving interpretability is essential for successful integration of radiomics models into clinical decision-support systems.

## 5 Integration of multi-omics and molecular imaging in tumor immunotherapy

Nuclear medicine imaging offers the advantage of evaluating organ and pathological tissue status from functional and metabolic perspectives, making it an essential tool in the field of oncology diagnosis and treatment. The integrated application of multi-omics and nuclear medicine imaging can further enhance the value of nuclear medicine imaging in tumor immunotherapy, providing stronger technical support for precise tumor status assessment and treatment decision-making guidance.

### 5.1 Integration of nuclear medicine imaging with genomics and transcriptomics

Nuclear medicine imaging enables non-invasive monitoring of tumor metabolism and functional status, while genomics and transcriptomics reveal the molecular genetic characteristics of tumors. The combination of these two approaches provides a more comprehensive analysis of tumor biological behavior and Immunotherapy response mechanisms. For instance, integrating 18F-FDG PET/CT imaging data with tumor genomics data can uncover correlations between metabolic parameters and gene mutations or gene expression profiles, offering new insights for biomarker screening. Studies have found that the SUVmax of 18F-FDG PET/CT is positively correlated with PD-L1 expression, which is regulated by multiple genes. This association suggests the potential for indirect prediction of PD-L1 expression and gene status through nuclear medicine imaging (Chu et al., 2021).

### 5.2 Integration of nuclear medicine imaging with proteomics and immunomics

Nuclear medicine immunoblotting is based on the principle of antigen-antibody specific binding, utilizing radionuclide-labeled specific antibodies to accurately display the size, location, extent of tumors and metastatic foci, as well as the expression status of immune-related molecules. When combined with proteomics and immunomics, it enables a deeper analysis of the immune characteristics of the tumor microenvironment and the response to immunotherapy. For instance, nuclear medicine probes targeting immune checkpoint molecules such as PD-1/PD-L1, CTLA-4, and LAG-3 can non-invasively monitor the expression levels and dynamic changes of these molecules. When integrated with the expression profiles of immune-related proteins detected by proteomics, this approach can more accurately identify patients who benefit from immune checkpoint inhibitor (ICI) therapy (McGranahan et al., 2016). The nuclear medicine probe targeting LAG-3 and PD-L1, developed by Professor He Yiyi’s team at Tongji University, has achieved visualization of lung cancer immunotyping and Immunotherapy resistance monitoring, providing a novel technical means for optimizing combined immunotherapy regimens.

### 5.3 Integration of nuclear medicine imaging and imagingomics

Nuclear medicine imaging provides rich functional and metabolic information for radiomics, while radiomics extracts quantitative features to uncover the latent value in nuclear medicine images. The integrated application of these two approaches significantly enhances the evaluation efficacy of nuclear medicine imaging in tumor immunotherapy. For instance, 18F-FDG PET/CT radiomics features can reflect tumor metabolic heterogeneity and immune cell infiltration status. When combined with qualitative assessments from nuclear medicine imaging, they enable more precise evaluation of immunotherapy efficacy and prognosis prediction (Prakadan et al., 2021). Additionally, radiomics models based on nuclear medicine imaging can predict specific immune response patterns, providing timely evidence for adjusting treatment decisions.

## 6 Summary and outlook

Tumor immunotherapy has become a cornerstone of modern cancer treatment; however, its precise implementation remains constrained by biological heterogeneity, dynamic tumor–immune interactions, and variability in therapeutic response. Multi-omics technologies provide a comprehensive framework for characterizing tumor genetics, immune microenvironment composition, metabolic reprogramming, and spatial heterogeneity, thereby enabling multidimensional stratification of patients and more accurate prediction of immunotherapy outcomes.

Among these modalities, radiomics offers a unique non-invasive bridge between molecular alterations and whole-tumor phenotypes. When integrated with genomic, transcriptomic, proteomic, and immunomic data, imaging-derived signatures can enhance risk stratification, therapeutic monitoring, and beneficiary population identification. The convergence of nuclear medicine imaging and multi-omics further expands the potential for dynamic and systemic immune evaluation.

To facilitate clinical translation, a structured roadmap is required: 1. multi-omics biomarker discovery in well-annotated cohorts; 2. cross-platform harmonization and multicenter validation; 3. development of standardized, interpretable predictive models; 4. regulatory evaluation and integration into clinical decision-support systems; and 5. continuous refinement through real-world clinical evidence. Standardization of data acquisition, model reproducibility, and explainability of artificial intelligence algorithms will be critical for large-scale implementation.

The increasing reliance on large-scale multi-omics datasets also raises important ethical and governance considerations. Data privacy protection, standardized data-sharing frameworks, and cross-institutional collaboration mechanisms are essential to ensure responsible implementation of precision oncology strategies.

Looking forward, the integration of artificial intelligence, multi-omics data, spatial biology, and real-world clinical datasets is expected to reshape precision immunotherapy. Such convergence may ultimately enable dynamic patient stratification, adaptive treatment optimization, and comprehensive immune monitoring, thereby improving long-term survival and therapeutic sustainability in cancer care.
